# Challenging a Misnomer? The Role of Inflammatory Pathways in Inflammatory Breast Cancer

**DOI:** 10.1155/2017/4754827

**Published:** 2017-05-14

**Authors:** Riley J. Morrow, Nima Etemadi, Belinda Yeo, Matthias Ernst

**Affiliations:** ^1^Olivia Newton-John Cancer Research Institute and La Trobe University School of Cancer Medicine, Heidelberg, VIC 3084, Australia; ^2^Austin Health, Heidelberg, VIC 3084, Australia

## Abstract

Inflammatory breast cancer is a rare, yet highly aggressive form of breast cancer, which accounts for less than 5% of all locally advanced presentations. The clinical presentation of inflammatory breast cancer often differs significantly from that of noninflammatory breast cancer; however, immunohistochemistry reveals few, if any, distinguishing features. The more aggressive triple-negative and HER2-positive breast cancer subtypes are overrepresented in inflammatory breast cancer compared with noninflammatory breast cancer, with a poorer prognosis in response to conventional therapies. Despite its name, there remains some controversy regarding the role of inflammation in inflammatory breast cancer. This review summarises the current molecular evidence suggesting that inflammatory signaling pathways are upregulated in this disease, including NF-*κ*B activation and excessive IL-6 production among others, which may provide an avenue for novel therapeutics. The role of the tumor microenvironment, through tumor-associated macrophages, infiltrating lymphocytes, and cancer stem cells is also discussed, suggesting that these tumor extrinsic factors may help account for the differences in behavior between inflammatory breast cancer and noninflammatory breast cancer. While there are various novel treatment strategies already underway in clinical trials, the need for further development of preclinical models of this rare but aggressive disease is paramount.

## 1. Introduction

Breast cancer is the most commonly diagnosed cancer among women, remaining a major cause of morbidity and mortality [1]. Patients who present with early or locally advanced disease are usually treated with curative intent by multimodality therapies. Inflammatory breast cancer (IBC) is a rare, yet highly aggressive variant of breast cancer, accounting for less than 5% of all locally advanced presentations [2]. The clinical presentation of IBC often differs significantly from that of noninflammatory breast cancer (non-IBC); however, immunohistochemistry reveals few, if any, distinguishing features [3]. Ultimately, while only accounting for a small proportion of diagnosed breast cancers, IBC remains responsible for 8–10% of all breast cancer-related deaths [4]. While significant progress has been made in the past decades in managing IBC, primarily through the introduction of multimodality treatment, survival rates remain incredibly poor, with approximately 40% of IBC patients alive three years postdiagnosis in contrast to 85% of non-IBC patients [5, 6].

## 2. Clinical Presentation and Treatment

**Figure 1:**
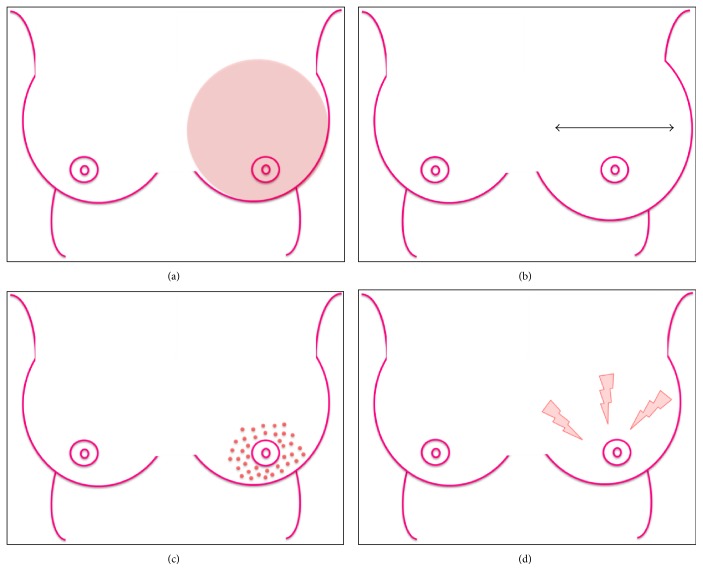
The common inflammatory-like symptoms that IBC patients present with, including (a) redness, (b) oedema, (c) skin dimpling, and (d) tenderness.

While the majority of patients with non-IBC present through breast cancer screening or with a breast lump, the clinical presentation of IBC differs substantially. Patients commonly present with inflammatory-like symptoms in the breast including erythema, oedema, tenderness, warmth, and/or skin dimpling [2, 6, 7] (Figure 1), which may be of rapid onset. However, less than half of patients with IBC present with a palpable breast lump [8–10]. The diagnosis of breast cancer in these patients is often delayed given that many of the clinical presentations mimic processes of infection, which then fail to respond to antibiotics. Collectively, these factors result in a majority of patients presenting with locally advanced disease (stage III) or with distant metastasis (stage IV) detected in approximately 30% of patients [6, 11, 12]. There are no specific radiological features on breast imaging which clearly differentiate IBC from non-IBC. Mammography may reveal breast enlargement, increased density, skin thickening, and less commonly multiple masses [9].

### 2.1. Histopathological Characteristics

While the clinical presentation defines IBC from non-IBC, pathological confirmation of invasive carcinoma is essential. Historically, the invasion of the surrounding dermal lymphatic system of the breast by disseminated tumor cells has been considered as a defining histological feature of IBC [13]; however, Bertucci and colleagues argued that this pathological hallmark is “neither mandatory nor sufficient for diagnosis” [14].

Luminal breast cancers are defined by their overexpression of estrogen receptor (ER) and/or progesterone receptor (PgR) and represent a more favorable breast cancer phenotype compared with hormone receptor-negative and HER2-positive breast cancers. A notable distinction between IBC and non-IBC is the overrepresentation of HER2-positive and triple-negative subtypes [9]. Numerous studies have reported that IBC tumors predominately exhibit downregulation of ER and PgR, a phenotype that shows a strong correlation with high-grade malignancy and shorter disease-free survival [6, 15]. For example, approximately 80% of non-IBCs are luminal compared with only half of all IBCs [4, 16]. In a multicenter retrospective analysis of 673 patients presenting with newly diagnosed IBC, 44% were ER positive, 34% PgR positive, and 26% had triple-negative breast cancer (TNBC) [10]. Furthermore, HER2-positive breast cancers represent approximately 20–25% of non-IBCs compared with up to 50% of IBCs [3, 9, 15]. As a result of these more aggressive phenotypes, metastatic spread, when present, often involves bone and soft tissue disease (e.g., the lung and liver). A significant number of patients with HER2-positive and triple-negative IBC present with central nervous system (CNS) relapse as their first site of relapse [10].

### 2.2. Current Treatments

Multimodality therapy should be offered to women with non-metastatic IBC. This may include a combination of surgery, radiotherapy, chemotherapy, endocrine therapy, and targeted therapy. Given the predominance of more aggressive phenotypes and larger tumor bulk at presentation, chemotherapy is often used in the neoadjuvant setting (prior to surgery) to assess response, or systemic therapy is used, to ideally downstage disease. When successful, this may convert a patient from inoperable to operable disease or improve the likelihood for surgical resection with clear resection margins [3]. HER2-targeted therapy with the use of trastuzumab, a humanized anti-HER2 monoclonal antibody, should be used for all HER2-positive patients, often in combination with chemotherapy. More recently from the NeoSphere study, which included 29 IBC patients, dual HER2 targeting with trastuzumab and pertuzumab, a second monoclonal antibody, in combination with chemotherapy in the neoadjuvant setting achieved significantly better pathological responses compared with those given with trastuzumab alone [17].

Response to neoadjuvant systemic therapy often guides the type of local therapy recommended for patients with non-IBC. However, due to its poor prognosis, surgical and radiotherapy decisions for IBC are usually based on the disease bulk at presentation. Hence, mastectomy remains the standard surgical treatment offered to patients with IBC regardless of clinical response to neoadjuvant therapy. Similarly, radiotherapy is usually administered regardless of the response to chemotherapy [3].

### 2.3. Prognosis

Prior to these combined interventions, 5-year survival rates for patients with IBC undergoing surgery and/or radiotherapy was less than 5% [13], and yet despite the implementation of modern multimodality treatments, prognosis for IBC patients remains poor. From a retrospective analysis of over 7600 patients diagnosed with IBC between 1990 and 2010, the short-term (two-year) breast cancer-specific survival was 71% with some improvement in survival identified for patients diagnosed in more recent years [18].  Table 1 summarises several of the larger and recently published IBC studies, which collectively suggest a long-term overall IBC patient survival rate of approximately 40%, highlighting the need for effective novel therapeutics for these patients. Even in HER2-positive IBC, the majority of patients will develop resistance to HER2-targeted therapies within a two-year period, which further complicates treatment [6, 15].

## 3. Molecular Characterization of IBC

In an attempt to molecularly characterize IBC based on clinical presentation as a distinct molecular entity, it became apparent that IBC comprised similar transcriptional heterogeneity as observed within non-IBC [12, 19]. Accordingly, the major molecular subtypes described for non-IBC also exist within IBC, with the associated prognostic and histological features mimicking that described in locally advanced breast cancers [12, 20].

Recently, Ross and colleagues used next-generation sequencing and comprehensive genomic profiling to identify potential therapeutic targets from metastatic lesions from the skin, chest wall, bone, liver, spine, and brain of 53 patients with relapsed IBC, of which 39% had TNBC and 25% had HER2-positive disease [21]. Among the most frequently mutated or amplified genes, these authors identified *TP53* and *MYC* as well as components of the RAS pathway and phosphoinositide 3-kinase (PI3K) pathways. Mutations of the RAS pathway included those encoded by the *ERBB2*, *KRAS*, *BRAF*, and *EGFR* genes, while in the PI3K pathway, *PIK3CA*, *PTEN*, *AKT1*, and *AKT3* were found to be mutated. This study highlights the diversity of genomic alterations occurring within IBC patients, indicating an opportunity for more personalized therapies that specifically target oncoproteins encoded by mutated or amplified genes.

### 3.1. Inflammation and IBC

Excessive abundance of cytokines and chemokines in the tumor microenvironment have become well-recognised factors that underpin the progression of solid malignancies. These soluble factors not only support survival, proliferation, and migration/invasion of tumor cells but also increase angiogenesis and facilitate the evasion of immune surveillance. Surprisingly, despite the clinical “inflammatory” features of IBC, relatively little is known about the role of these locally acting mediators, which are largely responsible for the communication between malignant cells and those that collectively make up the tumor stroma. A comprehensive study by Bieche and colleagues of 36 surgical IBC samples after neoadjuvant therapy analysed the expression of 538 genes implicated in tumor-associated inflammation and angiogenesis [19]. Surprisingly, these authors failed to detect any significant differences in the expression of many inflammatory cytokines and chemokines, including IFN-*γ*, TNF, IL-1*α*, IL-1*β*, IL-8, and IL-10 between IBC and non-IBC tissue samples, indicating that the associated inflammatory phenotype commonly visible is more likely due to blockage of the dermal lymphatics by disseminated tumor cells than by an infiltration of inflammatory cells [22]. However, while lymphatic vessels play an active role in the regulation of inflammatory processes [23], inflammation and its associated mediators have now been firmly established as drivers of tumor promotion. Despite the multitude of inflammatory mediators, the majority converge in malignant and normal cells at two shared intracellular signaling nodes. These comprise the latent transcription factor components of the nuclear factor kappa-light-chain-enhancer of activated B cells (NF-*κ*B) and signal transducer and activator of transcription- (STAT-) 3, with a lesser involvement of the RAS/mitogen-activated protein kinase (MAPK)/Jun kinase (JNK) pathway and cyclooxygenase (COX) enzymes [24]. Indeed, emerging evidence suggests that these pathways may also play an important role in IBC.

### 3.2. Inflammatory Signaling Pathways

#### 3.2.1. NF-*κ*B Pathway and Associated Cytokines

The NF-*κ*B family of transcription factors are well-documented critical players for cell survival, proliferation, immunity, and inflammation, thereby providing an important mediator for cancer emergence, progression, and metastasis [25]. The NF-*κ*B family consists of the five subunits RelA (P65), RelB, cRel, P105 (NF-*κ*B1), and P100 (NF-*κ*B2), which require dimerization in order to elicit transcriptional activity [26]. The activation of NF-*κ*B signaling can occur via classical (canonical) and alternative (noncanonical) pathways. Canonical NF-*κ*B pathway activation occurs in response to inflammatory stimuli, including TNF, IL-1*β*, and activators of toll-like receptors [27], while noncanonical NF-*κ*B activation occurs in response to ligand engagement of members of the TNF receptor superfamily, such as RANK, Fn14, lymphotoxin *β* receptor, and CD40 [28]. Canonical NF-*κ*B pathway modulates expression of genes involved in cell proliferation, survival, innate immunity, inflammation, and angiogenesis [29], while genes regulated by the noncanonical NF-*κ*B pathway regulate homeostasis of adaptive immunity and lymphangiogenesis [30–32].

In a cancer setting, tumors are often characterised by elevated levels of cytokines produced and secreted by the classical pathway, including two proinflammatory cytokines, TNF and IL-1*β*, which commonly trigger its activation [33]. Accordingly, constitutive activation of NF-*κ*B, which results in upregulation of antiapoptotic proteins, is frequently observed in ER-negative/HER2-positive tumors [25, 34–36]. Indeed, emerging evidence suggests that TNF fuels the progression of breast cancer by promoting proliferation, transformation, angiogenesis, invasion, and metastasis [37], as well as being vital for the viability of TNBC cell lines [38]. Conversely, HER2-positive breast cancer cells have been shown to acquire resistance to TNF-induced apoptosis [39]. The protumorigenic activity of TNF also extends to the luminal subtypes [40–42], and elevated TNF levels are associated with increased lymph node metastasis and advanced breast cancer stage [43].

Strikingly, ER-negative IBC tissues reveal excessive activation of many NF-*κ*B target genes compared with ER-negative non-IBC tissues [15, 20, 44]. Concordantly, Van Laere and colleagues also detected increased nuclear staining for the RelB and NF-*κ*B1 subunits and associated DNA binding [45, 46]. Hyperactivation of the MAPK signaling cascade is a common observation that also coincides with NF-*κ*B activation in IBC [9, 15]. Using the SUM-149 IBC cell line, it was observed that canonical and noncanonical NF-*κ*B pathway activity both promoted the formation of tumorspheres in vitro, suggesting that these pathways may regulate the function of tumor-initiating cells [47]. Furthermore, the inflammatory cytokines IL-6 and IL-8, which are among the best characterized NF-*κ*B target genes, are produced and secreted at high levels within IBC [6, 48].

While NF-*κ*B activation seems promising as a therapeutic target for IBC, there are currently no selective pharmacologic agents that target this pathway [15]. Furthermore, the critical role of NF-*κ*B in innate and adaptive immunity might be a barrier in inhibiting it long term. Therefore, inhibiting upstream inflammatory signals such as TNF, IL-17, and IL-1*β* could be a feasible strategy for IBC treatment, particularly in light of the wide use of the IL-1*β* antagonist anakinra, and TNF antagonists, etanercept and infliximab. An alternate and converse approach may exploit the presence of TNF in IBC to make this type of breast cancer a suitable candidate for a class of small molecule drugs designed to mimic the function of the second mitochondria-derived activator of caspases (SMAC/Diablo) [49]. These “SMAC-mimetics” inhibit the inhibitors of apoptosis proteins (IAPs), which positively regulate the canonical NF-*κ*B pathway. Thus, the inhibition of IAPs shifts the prosurvival signal of TNF towards the cell death pathway [50]. SMAC-mimetics also increase the sensitivity of some cancer cells to other TNF-related cell death stimuli, such as TRAIL and FasL. Indeed, Aird and colleagues suggested that IBC cells acquire resistance to anti-HER2 therapies or TRAIL due to the accumulation of X-linked IAP (XIAP) [51]. Accordingly, the SMAC-mimetic birinapant-induced cell death in the TRAIL-insensitive SUM-190 IBC cell line, while significantly increasing apoptosis in the TRAIL-sensitive SUM-149 cell line [52].

Within breast cancer, raised IL-1*β* levels are associated with poorly differentiated and more aggressive carcinomas [53]. In particular, overexpression of IL-1*β* has been identified within the serum of patients with ER-negative breast tumors [54]. Enhanced cell motility and invasion have also been coupled with overexpression of IL-1*β* within breast cancer via the upregulation of matrix metalloproteinase- (MMP-) 9, integrin-1, and E-selectin [55]. The secretion of IL-1*β* requires inflammasome activation and processing of pro IL-1*β* by Caspase-1 or 8 [56]. Inflammasome signaling becomes activated upon pathogen or danger-associated molecular patterns, or activation of programmed cell death machinery [57]. Streicher and colleagues showed that in the SUM-149 cell line, both IL-1*α* and *β* were expressed in an epidermal growth factor receptor- (EGFR-) dependent manner [55]. Autocrine expression of IL-1*α* and *β* in these cells induces NF-*κ*B activation, which is required for their proliferation and growth [55]. As expected, the secretion of IL-1*β*, unlike IL-1*α*, was not correlated with levels of mRNA, confirming the requirement of inflammasome activation. Likewise, the secretion of IL-1*α*, which occurs independently of inflammasome activation, was observed at high levels in the SUM-149 cell line. In addition, XIAP is an essential component of inflammasome signaling [58], and the presence of IL-1*β* in IBC can be associated to the previous observation of the accumulation of XIAP in IBC cells [59].

#### 3.2.2. JAK/STAT Pathway and Associated Cytokines

The Janus kinase (JAK) and STAT signaling pathway serves as major mediator for a wide variety of physiological responses to cytokines and growth factors during developmental and homeostatic processes [60, 61], including proliferation, survival, differentiation, metabolism, and apoptosis [62–64]. While a subset of the four members of the JAK family tend to be constitutively associated with growth factor and cytokine receptors, it appears that specific use of one of the 7 mammalian STAT proteins determines the biological outcome [65]. Thus, STAT3 and STAT5 primarily promote tumorgenesis, while STAT1 and STAT2, as part of the interferon response, play a major role during antitumor immune responses [66].

Fifty percent of primary breast cancers and breast cancer cell lines contain constitutive activation of STAT3 as indicated by its phosphorylation of a conserved tyrosine residue [63, 67]. Importantly, phosphorylated STAT3 (pSTAT3) is particularly abundant on the leading edge of tumors, as well as in surrounding lymphocytes and stromal cells, suggesting a role in invasion and metastasis [67]. Constitutive activation of STAT3 has also shown to accelerate tumor progression and increase the metastatic potential in HER2-positive breast cancers [68].

In tumorspheres derived from the SUM-149 IBC cell line, cell death is induced following the administration of a novel JAK2 inhibitor, with associated STAT3 inhibition [69]. This coincides with findings from other investigators that the JAK2/STAT3 pathway appears necessary for propagation of the cancer stem cell (CSC) phenotype of SUM-149 cells [70]. A retrospective study conducted by Jhaveri and colleagues analysed the baseline expression of JAK/STAT signaling pathway components in IBC by immunohistochemical analysis [48]. Extensive expression of IL-6 and the activated isoforms pJAK2 and pSTAT3 were observed within IBC, as well as in non-IBC after treatment with chemotherapy. Multiple genomic studies on both primary cell lines and tumor specimens have confirmed excessive IL-6 production and secretion as a hallmark of IBC [2, 7, 19].

Serum IL-6 concentrations are not only increased in more than half of all breast cancer patients [71] but are also significantly higher in IBC patients compared to non-IBC patients [72]. Similar to other solid malignancies, elevated IL-6 expression in breast cancer positively correlates with increased tumor stage, lymph node involvement, recurrence risk, and distant metastasis [67]. These findings may not surprise given the extensive in vitro studies suggesting that IL-6 promotes proliferation, induces changes in morphology, and regulates cell adhesion [73–75]. IL-6 also promotes self-renewal of the stem cell characteristics of the IBC cell lines SUM-149 and SUM-190 [70], although this is thought to occur by induction of cytokine expression by IBC cells themselves, which result in autocrine stimulation of Notch signaling [6, 70]. Indeed, mRNA analysis has also confirmed IL-6 receptor expression in IBC cells [73, Morrow unpublished observations], and IL-6/STAT3 signaling has been proposed to underpin the dynamic equilibrium between stem and nonstem breast cancer cells [76]. Thus, IL-6 may play a similar role by propagating a CSC-like phenotype in IBC [15].

IL-6 expression in SUM-149 cells has also been associated with direct regulation of the Ras homolog gene family member C- (RhoC-) GTPase, which is highly expressed in IBC tumors compared with stage-matched, non-IBC tumors [2, 15, 77]. Elevated RhoC-GTPase activity enhances the metastatic potential of IBC cells by affecting the cytoskeleton and altering cellular adhesion to the extracellular matrix [6, 9].

One major implication of IL-6 in breast cancer relates to observations that the acquisition of drug resistance occurs within cell populations that are able to produce higher levels of the cytokine [78]. Similarly, it has been observed that failure of HER2 and other directed therapies coincides with IL-6-mediated activation of STAT3 [79–81]. IL-6-dependent activation of the JAK2/STAT3/calprotectin axis has shown promise as a therapeutic target for hormone receptor-negative and HER2-positive breast cancers, as these subtypes produce higher levels of IL-6 [80]. This signaling cascade was found to be prevalent for the viability of the IBC SUM-190 cell line, with tumorigenicity reduced in vitro and in vivo when targeted with FDA-approved inhibitors, alone and in combination with HER2 inhibitors [80].

#### 3.2.3. EGFR/PI3K/mTOR Pathway and Associated Cytokines

Recent developments in molecular therapies for HER2-positive breast cancers have been highly successful using monoclonal antibodies targeting the receptor (i.e., trastuzumab), or small molecules inhibiting the receptor-associated kinase (i.e., lapatinib). Preclinical studies have been undertaken to establish the benefits of targeting the activity of RhoC, EGFR, and P27KIP for the treatment of IBC [82]. Accordingly, small-interfering RNA-targeting EGFR or inhibitors of the enzymatic activity of EGFR reduce the invasion of SUM-149 cells in vitro [83]. Likewise, knockdown of HER2 and EGFR in IBC cell lines decreased their colony growth in soft agar and increased caspase activation following extracellular matrix detachment via the activation of the ERK/MAPK signaling pathway [84]. These observations suggest a mechanism by which targeting EGFR/HER2 and associated RAS/ERK pathways may confer a therapeutic benefit by inducing anoikis in IBC cells.

The PI3K/AKT and mammalian target of rapamycin (mTOR) pathways are not only commonly regarded as a single overlapping pathway but are also crucial regulators of growth, survival, proliferation, and metabolism of cancer cells. Accordingly, excessive activity of the PI3K/mTOR pathway is associated with acquired resistance in non-IBC to targeted (i.e., endocrine and HER2 directed), as well as cytotoxic therapies [85]. As outlined above, genomic abnormalities in the HER2/PI3K/mTOR pathway are frequently observed in IBC. Immunohistochemical analysis of phosphorylated ribosomal protein-S6 (pS6), a marker of mTOR pathway activation, revealed strong expression (2+ or greater) in 95% of IBC tumor specimens, as well as in residual tissue after neoadjuvant chemotherapy in patients with non-IBC, suggesting a possible mechanism of therapeutic resistance [48]. Interestingly, IBC had less pathway activation in surrounding nontumor tissue, which may allow for more specific therapeutic effects to be delivered to cancer cells. Strikingly, Jhaveri and colleagues also showed that of those IBC patients positive for pS6, 95% showed strong activation (2+ or greater) for JAK2, highlighting the cross activation between inflammatory signaling pathways, which may have important therapeutic implications [48].

The PI3K/Akt and P38 pathways are also activated in response to engagement of the cognate receptors CXCR1 and CXCR2 for IL-8. IL-8 is a chemokine produced in response to toll-like receptor activation by macrophages, epithelial cells, and airway smooth and muscle cells and serves as a neutrophil chemotactic factor [86, 87]. IL-8 promotes breast cancer progression by increasing cell invasion, angiogenesis, and metastasis and is upregulated in HER2-positive breast cancer [87], while also being involved in promoting expansion of breast CSCs [87]. In comparison to non-IBC, IL-8 alongside CXCR1/2 is expressed more markedly in IBC cell lines and tumor tissues in comparison to non-IBC [20, 45]. Moreover, in vitro models have determined that IL-8 secreted from human monocytes can promote invasion and motility of IBC cells through stimulation of the PI3K/AKT signaling cascade [88]. In line with the aforementioned, and similar to IL-6, IL-8 secreted from mesenchymal stem cells (MSCs) can stimulate the CSC self-renewal of SUM-149 cells [70]. Given the complementing cellular sources for IL-8 production, comprising cells of the tumor as well as of its microenvironment, targeted interference with IL-8 signaling may provide therapeutic benefit, particularly in HER2-positive disease.

#### 3.2.4. COX Pathway

The cyclooxygenase (COX) family of enzymes comprises COX-1 and COX-2, which differ in their tissue distribution and function [89]. Collectively, these enzymes catalyse the conversion of arachidonic acid to prostaglandins, which are mediators of adhesion, growth, and differentiation. Expression of COX-1 is common in many tissue and cell types, while COX-2 is induced only in response to proinflammatory stimuli. Most of these stimuli promote COX-2 expression via protein kinase C and RAS-dependent signaling.

Overexpression and aberrant activation of COX-2 have been identified in a range of solid malignancies, including the colon, prostate, pancreatic, and bladder [89]. Likewise, levels of COX enzymes are increased within 40% of breast cancers [90], while prostaglandin E2, the main product catalysed by COX-2, is produced at high levels in various human breast cancer cell lines [91, 92]. Expression of COX-2 has been confirmed in 13 breast cancer cell lines by qRT-PCR, with no detection in normal breast tissue [93]. COX-2 expression also correlates with poor prognostic indicators, such as increased tumor size, axillary node and distant metastasis, tumor grade, high-proliferation rates, receptor-negative disease, and HER2 amplification [94, 95]. The molecular IBC signature defined by Van Laere and colleagues suggested elevated COX-2 expression in IBC compared to non-IBC tumors, and this was also reflected by more abundant prostaglandin E2 in primary and metastatic IBC tumors [20, 45, 96]. Furthermore, suppression of prostaglandin E2 binding to its cognate receptors inhibited the aggressive proliferation and invasion of the SUM-149 cell line in vitro [96].

## 4. Inflammatory Cell Types and Cellular Processes in IBC

**Figure 2:**
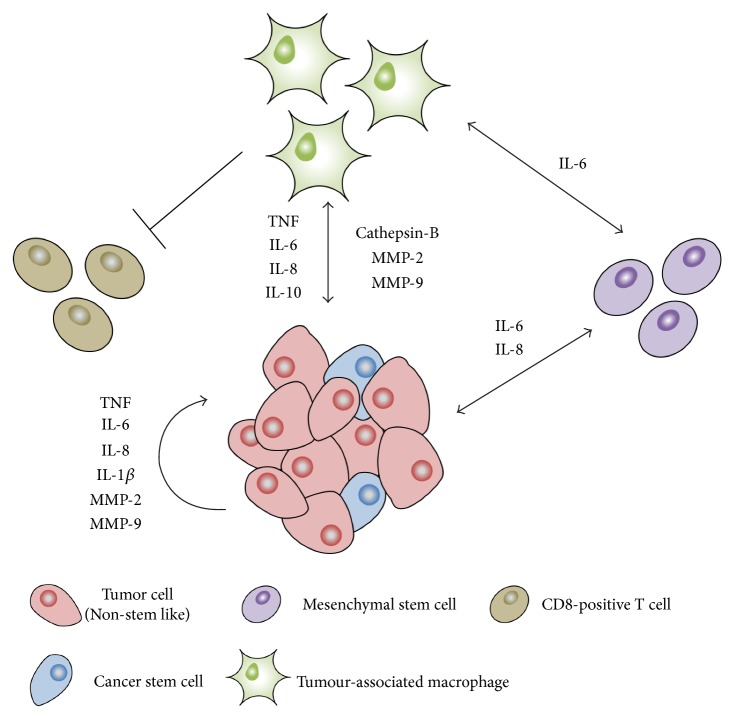
Schematic representation of important inflammatory pathways underpinning communication between IBC cells (tumor cells and cancer stem cells) and major cell types constituting the tumor microenvironment (mesenchymal stem cells, tumor-associated macrophages, and CD8-positive T cells are shown). Arrows indicate the cross talk of inflammatory mediators secreted by the indicated cell types to promote IBC progression.

Breast cancers arise from multifactorial and dynamic processes and interplay between neoplastic cancer cells with driver mutations and a plethora of genetically stable cell types that collectively account for the tumor microenvironment. Accordingly, carcinomas are heavily infiltrated by various types of macrophages/monocytes, lymphocytes, and leukocytes, alongside extracellular matrix-depositing fibroblasts and other stromal cell types (Figure 2). Coerced by cancer cells to support their growth, tumor-associated stromal cells provide a regulatory network that can modify the phenotype of cancer cells to confer acquisition of a stem cell phenotype, resistant to drug treatment. Indeed, it has been speculated that the hypersensitivity of IBC to tumor extrinsic factors may account for the differences in behavior between IBC and non-IBCs and that a limited focus on tumor intrinsic gene expression features may not fully explain the aggressive nature of IBC.

### 4.1. Tumor-Associated Macrophages/Monocytes

Macrophages are established as a major inflammatory cell type, which depending on their endotype, either control cancers by displaying their phagocytic phenotype or promote tumor progression when polarized towards a wound-healing/angiogenic phenotype. The latter is the prevalent endotype of macrophage infiltrates in breast carcinomas [97, 98]. These tumor-associated macrophages (TAMs) produce and secrete high levels of inflammatory mediators that not only promote survival and proliferation of neoplastic cells but also antagonize the antitumor activity of CD8-positive T cells [99, 100]. Studies have confirmed that IBC tumors show high infiltration of TAMs [48]. Wolfe and colleagues described the accumulation of CD68-positive macrophages in the normal tissue surrounding IBC lesions [101].

Considering TAMs produce matrix-degrading enzymes, these cells also play a critical role in the dissemination and spread of breast cancers, resulting in a positive correlation in IBC between infiltration of macrophages, lymph node metastasis, and expression/activation of proteases including cathepsin-B and MMPs-2/9 [102, 103]. Thus, TAMs also contribute to the metastasis of IBC cells via releasing mediators of invasion and angiogenesis including TNF, IL-6, IL-8, and IL-10. Collectively, the expression of these cytokines is significantly higher in CD14-positive tumor-infiltrating monocytes of IBC patients than in those from non-IBC patients [104].

### 4.2. Tumor-Infiltrating Lymphocytes

The presence of tumor-infiltrating lymphocytes and in particular, the proportion of functional cytotoxic CD8-positive T cells have been suggested to predict patient response to immune checkpoint treatment. In contrast, the presence of exhausted T cells with poor effector function is typically associated with the expression of programmed death-ligand 1 (PD-L1) and other immune checkpoint inhibitors on tumor cells and/or tumor-infiltrating lymphocytes [105]. Thus, high PD-L1 expression may negatively regulate T cells, thus preventing the activation and migration of CD8-positive T cells into IBC tumors.

There has been rapid expansion of phase II and III clinical trials of immunotherapy for patients with metastatic breast cancer, predominantly in triple-negative and HER2-positive disease. Given the overrepresentation of these phenotypes in IBC, their inclusion into such trials is paramount to identify whether an immune approach offers substantial survival gains (e.g., NCT02411656). Bertucci and colleagues analysed mRNA expression of PD-L1 in over 300 IBC tumor samples and identified higher expression in 38% of IBC tumors compared to normal breast tissue, which was associated with a better response to chemotherapy [106].

IHC staining identified aggregates of CD8-positive T cells as major subpopulations associated with intratumoral and peritumoral desmoplastic stroma in approximately half of IBC tumors analysed, with low density of single-spread cells across other samples [107]. However, these tumors stained minimally for the regulatory T cell marker FoxP3, while tumor-associated staining of the immune checkpoint regulator PD-L1 varied greatly. Not surprisingly, the same authors observed a positive correlation between the extent of CD8-positive T cell infiltration and mutation rate as a predictor of variability of neoantigen, which in turn correlated with the presence of several mutations in gene-encoding DNA mismatch repair genes.

### 4.3. Mesenchymal Stem Cells

MSCs are a heterogeneous subset of multipotent progenitor cells capable of differentiating along multiple cell lineages, including into osteoblasts, chondrocytes, and adipocytes [108, 109]. Tumor cells are able to selectively recruit MSCs to the primary and metastatic sites, where they form tumor stroma and alter the tumor microenvironment, facilitating the growth and spread of a number of cancers [110]. Indeed, MSCs derived from breast cancer tissue are capable of promoting proliferation and migration of breast cancer cells [111–113].

Interestingly, conditioned media from MSCs allow for mammosphere formation of the IBC cell lines SUM-149 and MDA-IBC3 via decreasing the expression of E-cadherin and increasing the expression of other epithelial-to-mesenchymal- (EMT-) related proteins, such as N-cadherin, vimentin, and fibronectin [114]. Coinjection of MSCs with MDA-IBC3 cells in vivo has also shown to shorten the latency period for tumor initiation [115].

Wolfe and colleagues also demonstrated in a SUM-149 xenograft model that M2-polarised macrophages are essential for the MSC promotion of skin invasion, which is dependent on IL-6 signaling [101]. Considering this, the inhibition of colony-stimulating factor-1, a key cytokine for activation and recruitment of macrophages, showed a reduction in the growth of SUM-149 tumors, skin invasion, and local occurrence, as well as a reduction in the percentage of infiltrating TAMs. Further, pSTAT3 levels were reduced, indicating that inhibiting the recruitment of macrophages may reduce IL-6 signaling between MSCs and IBC cells. Therapeutically, specific targeting of M2 macrophages in IBC patients through the use of statins may yield interesting findings.

### 4.4. Cancer Stem Cells

CSCs are a subpopulation of cells that exhibit characteristics similar to embryonic stem cells, in that they are capable of limitless replication and multipotency, thereby endowed with the capacity to give rise to various differentiated cells that contribute to tumor heterogeneity [116, 117]. Recently, compelling evidence suggests that CSCs support and stimulate protumorigenic characteristics of cancer and of stromal cells that collectively allow for breast cancer progression [118]. CSCs have also been shown to undergo EMT, another protumorigenic feature that further links them with tumor progression [119]. Indeed, non-IBC cells that undergo EMT acquire CSC-like characteristics [120, 121].

IBC cells seem to adopt a more CSC-like phenotype, which may contribute to the aggressive and motile characteristics of IBC [96]. In particular, SUM-149 cells display CD44^+^/CD24^−/low^ stem cell surface markers, as well as aldehyde dehydrogenase-1, a maker of tumor initiation [122]. This phenotype is also observed within CSCs, which when injected into mice, are highly tumorigenic [15]. Likewise, a patient-derived xenograft of IBC (MARY-X) also exhibits this phenotype, along with the unique stem cell marker CD133 [123]. Therapeutically, targeting of this CSC phenotype within IBC cells via Notch pathway inhibition has a significant reduction in anchorage independent growth of SUM-190 and SUM-149 cells [70].

### 4.5. Angiogenesis

IBC is highly angiogenic, with biopsies often characterized by increased microvessel density when compared to non-IBC [124, 125]. Accordingly, neoadjuvant treatment of treatment-naïve patients with primary IBC with angiogenesis inhibitors (i.e., bevacizumab and trastuzumab) and chemotherapy is efficacious and well tolerated in patients with previously untreated primary IBC [126]. However, a subsequent study suggested that the addition of bevacizumab to neoadjuvant and adjuvant chemotherapy did not provide clinical benefit to patients with nonmetastatic HER2-negative IBC [127]. On the other hand, molecular targets for lymphangiogenesis and vasculogenesis have demonstrated greater potential in IBC than in non-IBC, as a consequence of IBC showing rapid accumulation of cancerous cells in the dermal lymphatic system, rather than extensive primary tumor formation [96, 125, 128–130]. Given that normal epithelial cells require attachment to the extracellular matrix for survival, a comprehensive understanding of the molecular mechanisms underlying IBC cell survival in the lymphatic vessels is likely to shed light on new therapeutic opportunities, including regulators of anoikis.

### 4.6. Epithelial-to-Mesenchymal Transition

An integral part of the metastatic process involves EMT, where cancer cells gain motility and stem cell-like capabilities. Perhaps counter-intuitively, gene expression profiling of IBC samples revealed no clear evidence for EMT [14]. One reason underpinning these findings is the observation that, although EMT in IBC includes expression of stem cell markers alongside *FN1*, *VIM*, *TGM2*, *ZEB1*, and other regulators of a mesenchymal fate, it is also associated with increased expression of the epithelial adhesion molecule E-cadherin [131]. While these observations appear to be unique to IBC, and possibly explain the higher presence of tumor microemboli formation, EMT in most other epithelial systems is correlated with reduced E-cadherin expression. Instead, emerging evidence strongly suggests that activated immune cells within the tumor microenvironment promote EMT in IBC cells by secreting proinflammatory cytokines including TNF, IL6, and TGF-*β* [132].

## 5. Novel Therapeutic Targets of IBC

Despite recent insights suggesting potential for targeted therapies, a majority of the current treatment regiments for IBC still provide significantly inferior survival outcomes for patients. This is illustrated by the currently recruiting trials for IBC (Table 2), which predominantly use chemotherapy as the “back bone” for treatment.

The recently published BEVERLY-1 phase II trial investigated the combination of the antiangiogenic bevacizumab in the neoadjuvant setting with conventional chemotherapy and determined the pathological complete response to be only 19% in a cohort of 100 patients [127]. From this result, the authors concluded that bevacizumab did not provide any additional clinical benefit.

Several small molecule inhibitors have been investigated in IBC. The tyrosine kinase inhibitor lapatinib has been extensively investigated and approved for use in HER2-positive metastatic breast cancer. Accordingly, lapatinib was assessed in a neoadjuvant setting in combination with weekly paclitaxel in both HER2 positive as well as HER2-negative/EGFR-positive IBC [133]. Due to poor accrual of the HER2-negative/EGFR-positive cohort, only the HER2-positive cohort was analysed, showing a pathological complete response rate of 18.2%. A phase II study of afatinib, which inhibits both HER2 and EGFR receptors, recruited IBC patients with HER2-positive disease to either afatinib monotherapy or in combination with the chemotherapy drug vinorelbine (NCT01325428). Thirty-five percent (9 of 26) and twenty percent (2 of 10) of patients had clinical benefit from being treated with afatinib monotherapy and afatinib plus vinorelbine, respectively [134].

Inhibitors targeting various components of the IL-6/JAK/STAT3 pathway have been effective in a number of preclinical models of breast cancer. Ruxolitinib, a dual JAK1/2 inhibitor, reduces STAT3 activation in a range of TNBC cell lines [33]. Considering its efficacy in myelofibrosis [135], ruxolitinib is now undergoing clinical trials on a range of breast cancer subtypes. One such trial is currently in phase II for the treatment of patients with recurrent, metastatic, or triple-negative IBC (NCT02041429). Another phase II trial for breast cancer patients, including IBC, focuses on disease with evidence of pSTAT3 (NCT01562873). Complementing studies with other JAK2 inhibitors reduced STAT3 activity and induced cell death in SUM-149 cells [69]. Further, an IL-6 receptor-targeting antibody, tocilizumab, that shows great efficacy in preventing IL-6 signaling and is FDA approved for the treatment of patients with rheumatoid arthritis [136, 137] is only beginning to be assessed as a therapeutic option within IBC. Recently, tocilizumab was shown to compromise the viability of ER-negative/HER2-positive IBC cells, with a more potent effect observed when used in combination with anti-HER2 therapies [80].

To date, there are several factors that limit the availability of trials for IBC patients, which include the relative scarcity of preclinical IBC models upon which to test novel therapeutics prior to introduction to early phase trials. The literature is dominated by retrospective series of patients with IBC with only archival tissue available for research. In addition, patients with IBC are often excluded from large phase II and III clinical trials of novel therapeutics. Given the rare presentation of IBC, trials designed specifically for these patients often fail to achieve their target accrual. A combined, collaborative effort is needed to identify these patients and enable access to fresh tissue (ideally pre- and postsystemic treatment) for the generation of patient-derived xenograft models, which can be utilised by translational scientists to better understand IBC and to develop and test new and more effective therapies to improve long-term survival.

## 6. Conclusion

Despite mounting evidence suggesting that inflammatory processes are contributing to the development, progression, and maintenance of IBC, it remains uncertain whether the IBC nomenclature is the most adequate terminology to describe this clinically distinct entity of aggressive breast cancer. Multiple inflammatory signaling cascades have been identified as deregulated and/or overactive within IBC lesions, contributing to inflammatory loops being established between IBC tumor cells and cells of the surrounding tumor microenvironment. Importantly, these findings are beginning to be translated into a clinical setting to assess whether previously designed and tested therapeutics in a non-IBC setting also function as effectively within IBC. However, caution should be taken when interpreting findings on IBC samples, as many places focus upon analysing RNA expression, which of course does not reflect the functional protein level.

Essential to our understanding of IBC as a disease is the establishment of additional preclinical models, which will allow us to explore and confirm with greater confidence whether inflammatory processes are indeed contributing to the progression of IBC more than in a non-IBC setting. Further, the rarity of IBC human tissue samples needs to be appreciated more than it currently is. Acquiring pretreated tissue from IBC patients is vital for furthering our understanding of the biochemical modifications occurring that allow for the progression of IBC, through increasing invasion, avoiding immune surveillance, and resisting therapeutics. Ultimately, therapeutically targeting multiple levels of a range of inflammatory signaling cascades seem a rational and attractable avenue to explore within IBC.

## Figures and Tables

**Table 1:** Largest and recent retrospective series of patients with IBC. Abbreviations are as follows: *N*, number of patients included in the series; RT, radiotherapy; LRFI, local recurrence-free interval; DFI, disease-free interval; DDFI, distant disease-free interval; OS, overall survival; pCR, pathological complete response; HR+ve, hormone receptor positive; HER2−ve, HER2 negative; LRFS, local relapse-free survival; Mx, mastectomy; BCSS, breast cancer-specific survival; TNBC, triple-negative breast cancer; NAC, neoadjuvant chemotherapy; CT, chemotherapy.

Reference	Study population	*N*	Outcome
Abrous-Anane et al. 2011 [138]	Single institution, IBC patients 1985–1999; all received NAC followed by either surgery, RT, or surgery + RT. All patients received anthracycline-based chemotherapy but only 16% received a taxane.	232	At 10 years follow-up, LRFI was 78% surgery versus 59% radiotherapy-only group; DFI 26%, DDFI 37% and OS 38%. No significant differences in OS, DFI, or DDFI between the exclusive RT and surgery groups (*p* = 0.32, *p* = 0.35, and *p* = 1, respectively).
Masuda et al. 2014 [139]	MD Anderson series, IBC patients 1989–2011, all received NAC. 17% received anthracycline; 72% anthracycline + taxane, 3% taxane, 9% taxane + trastuzumab. 55% of HER2+ve patients received trastuzumab. 86% received neoadjuvant and/or adjuvant RT.	527	pCR rate (stage III IBC) 15.2% (only 7.5% in HR+ve, HER2−ve while 30.6% in HR−ve, HER2+ve); TNBC worst survival. Factors associated longer DFS and OS: pCR, no evidence of vascular invasion, non-TNBC, adjuvant hormonal therapy, RT.
Panades et al. 2005 [140]	British Columbia series, IBC patients 1980–2000.	485	Among patients treated with curative intent, median BCSS 3.2 years; 10 yr LRFS for patients having Mx after CT, Mx before CT, and without Mx was 62.8%, 58.6%, and 34.4%, respectively (*p* = 0.0001); 10 year BCSS was 36.9%, 19.9%, and 22.5%, respectively (*p* = 0.005).
Do Nascimento et al. 2016 [141]	Single institution, IBC patients 2001–2010; 41% HER2+ve, 18% TNBC; 77% had early disease at diagnosis.	57	35/44 underwent surgery and 16 are relapse-free. 6/44 achieved pCR; median survival in 13 patients with metastatic disease at diagnosis was 21.7 months.
Bonev et al. 2014 [142]	Single institution, IBC patients 2002–2006 receiving NAC (AC-T) + trastuzumab (if HER2+ve) + bevacizumab (if HER2−ve).	24	29% partial Mx and 71% Mx. OS partial mastectomy 59% and for Mx 57% (*p* = 0.49), respectively, at a median follow-up of 60 months.
Gogia et al. 2014 [143]	Single institution India, 2004–2012; stages III and IV. All nonmetastatic IBC patients received anthracycline and/ or taxane-based chemotherapy followed by modified radical Mx, RT. No trastuzumab.	41	pCR 15%. At a median follow-up of 30 months, the 3-year relapse-free survival 30% and OS 40%.
Matro et al. 2015 [10]	Multi-institutional study, IBC patients 1999–2009; 29% had metastatic disease at presentation.	673	Median survival 66 months for stage III and 26 months for stage IV. Among 82% of stage III patients receiving multimodality therapy, the median survival was 107 months.

**Table 2:** Recent recruiting trials in IBC. Abbreviations are as follows: T, tumor stage; N, nodal; FEC, fluorouracil epirubucin cyclophosphamide; pCR, pathological complete response; MTD, maximum tolerated dose; PFS, progression-free survival; DCR, disease control rate; PD, progressive disease.

Trial name	Study population	Phase	Study target	Treatment arms	Primary outcome
NCT01880385	T4d, any N stage, IBC	I	30	Open-label neoadjuvant bevacizumab + FEC followed by adjuvant docetaxel (+/−trastuzumab in HER2 positive) and RT	pCR
NCT02623972	HER2-negative, locally advanced IBC	II	25	Neoadjuvant eribulin followed by doxorubicin and cyclophosphamide	pCR
NCT01938833	Metastatic, HER2-negative IBC	I/II	47	Romidepsin and Nab-paclitaxel until PD or unacceptable toxicity	MTD and PFS
NCT02389764	Metastatic, HER2-negative IBC	II	44	Oral nintedanib	CBR
NCT00820547	T4d, any N (stage IIIB or IIIC), HER2-negative IBC	II	100	Neoadjuvant FEC + bevacizumab followed by adjuvant docetaxel + 12 months bevacizumab	pCR
NCT02411656	HER2-negative, metastatic IBC or recurrent disease after treated primary	II	35	Adjuvant pembrolizumab for up to 24 months	DCR
NCT01036087	HER2-negative, locally advanced IBC	II	40	Neoadjuvant panitumumab + nab-paclitaxel + carboplatin + FEC	pCR
NCT01796197	Nonmetastatic, HER2-positive IBC	II	30	Preoperative trastuzumab + pertuzumab + paclitaxel followed by adjuvant trastuzumab + pertuzumab +/− AC	pCR
NCT02041429	Unresectable or metastatic triple-negative IBC	I/II	24	Preoperative ruxolitinib + paclitaxel	MTD
NCT01525966	Locally advanced, triple-negative IBC (also open in non-IBC)	II	69	Preoperative carboplatin and paclitaxel (albumin-stabilized nanoparticle)	pCR
